# Adult Hirschsprung disease: salvage to total colectomy – a rare case report

**DOI:** 10.1093/jscr/rjag519

**Published:** 2026-06-26

**Authors:** Saurav Poudel, Sachina Belbase, Kunal Bikram Deo, Anusha Bhattarai, Rashmita Bhandari, Shivesh Batajoo

**Affiliations:** Department of Gastrosurgery, Nobel Medical College Teaching Hospital, Kanchanbari, Morang, Biratnagar-4, 56113, Nepal; Department of Gastrosurgery, Nobel Medical College Teaching Hospital, Kanchanbari, Morang, Biratnagar-4, 56113, Nepal; Department of Gastrosurgery, Nobel Medical College Teaching Hospital, Kanchanbari, Morang, Biratnagar-4, 56113, Nepal; Department of Gastrosurgery, Nobel Medical College Teaching Hospital, Kanchanbari, Morang, Biratnagar-4, 56113, Nepal; Department of Pathology, Nobel Medical College Teaching Hospital, Kanchanbari, Morang, Biratnagar-4, 56113, Nepal; Department of Gastrosurgery, Nobel Medical College Teaching Hospital, Kanchanbari, Morang, Biratnagar-4, 56113, Nepal

**Keywords:** adult Hirschsprung’s disease, gastrointestinal surgery, recurrent constipation, staged surgical procedures

## Abstract

A 17-year-old male with chronic constipation presented with acute obstipation and abdominal distension. Computed tomography showed a dilated colon with rectosigmoid transition. Initial laparotomy and segmental resection confirmed Hirschsprung disease (HD) on histopathology. One month later, he re-presented with abdominal distension. Urgent total colectomy and ileostomy were performed. He recovered after ICU care and was discharged with a functioning ileostomy. This case underscores complications of incomplete resection in adult HD and the need for vigilant follow-up.

## Introduction

Hirschsprung disease (HD) in adults is rare and frequently misdiagnosed as chronic idiopathic constipation. It results from the absence of ganglion cells, mainly in the rectum and distal sigmoid colon, and is usually diagnosed before age 5 [1]. Barium enema may show smooth narrowing with proximal dilatation (83% sensitivity), but biopsy confirmation of aganglionosis is essential [2]. Long-term issues include persistent bowel dysfunction, fertility problems, and impaired urological/sexual function, adversely affecting psychosocial well-being [3]. We report a 17-year-old male with adult HD.

## Case report

A 17-year-old male presented with difficulty passing stool for 3 months, obstipation for 2 days, anorexia for 10 days, and abdominal pain for 10 days. He gave a history of constipation since infancy, requiring regular laxatives, enemas, and occasional manual disimpaction. There was no history of delayed meconium passage or failure to thrive. On examination, the vitals were stable, but the abdomen was distended and non-tender with diffuse swelling in the left lower quadrant. Laboratory findings included hemoglobin 5.6 g/dl, white blood cell (WBC) count 5800/cu mm (60% neutrophils), peripheral blood smear showing marked microcytic hypochromic anemia, elevated amylase at 127 U/L, elevated C-reactive protein (CRP) at 132 mg/L, and elevated lactate at 2.2 mmol/L.

**Figure 1:**
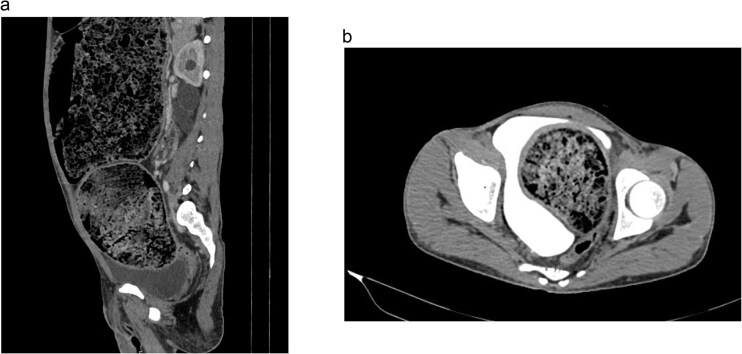
(a and b) Dilated transverse, descending and sigmoid colon in CT-abdomen.

Ultrasound revealed bilateral hydronephrosis with a gaseous abdomen. Contrast-enhanced computed tomography (CT) abdomen demonstrated markedly dilated, fecal-loaded transverse, descending, and sigmoid colon with an abrupt transition at the rectosigmoid junction, along with bilateral hydronephrosis and mild ascites (Fig. 1a and b). A presumptive diagnosis of colonic obstruction was made. The colon with its mass effect and without acute presentation in childhood, sarcoma, or other tumors was considered as differentials.

**Figure 2:**
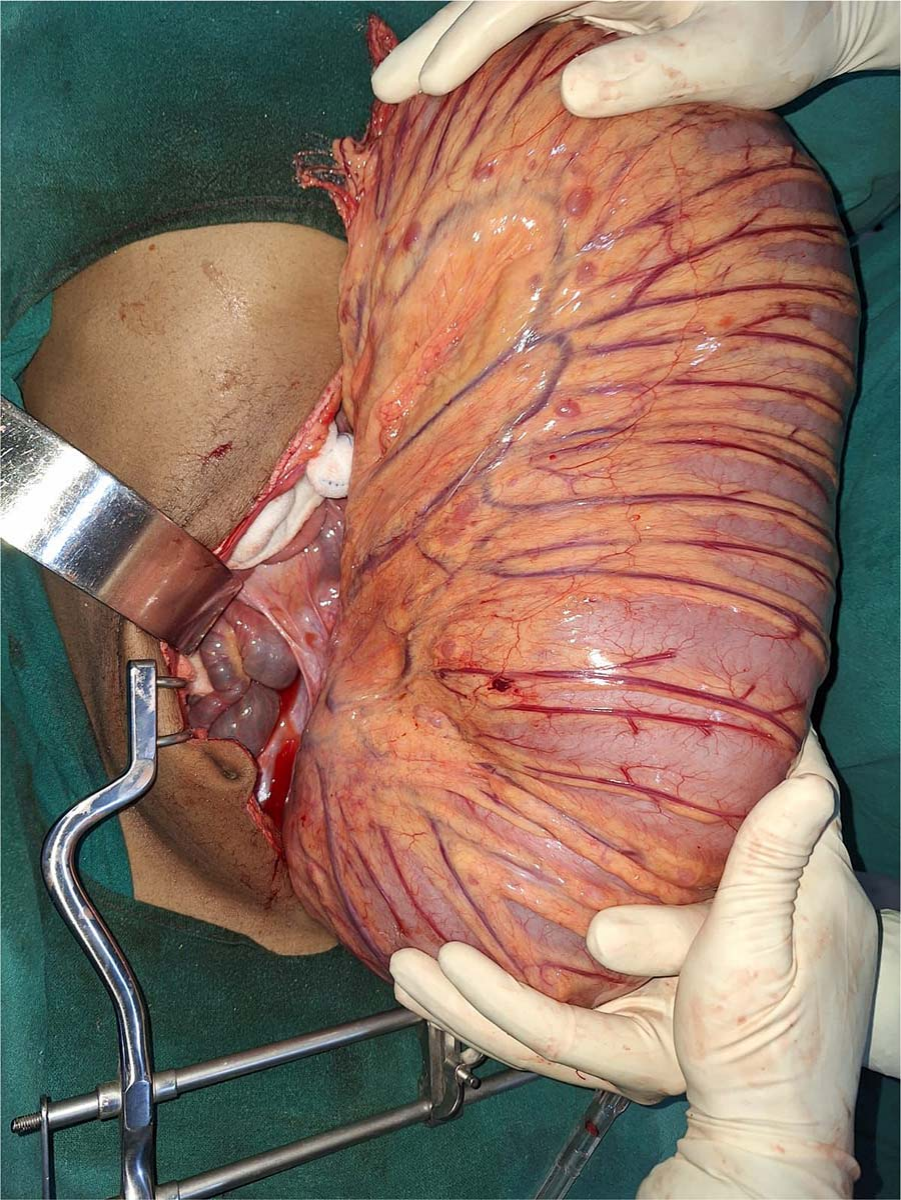
Massively dilated colon visible after exploratory laparotomy.

Exploratory laparotomy was performed, revealing a largely dilated sigmoid colon (~ 15 cm) with fecal loading, a dilated descending colon (~ 25 cm), and transverse colon dilatation with fecal loading (Fig. 2). Left hemicolectomy, sigmoidectomy with anterior resection of rectum, end transverse colostomy, and Hartmann’s closure of the rectal stump were performed. A conservative approach was adopted to ensure the colon’s continued function. The transitional section was sent for histopathological examination.

**Figure 3:**
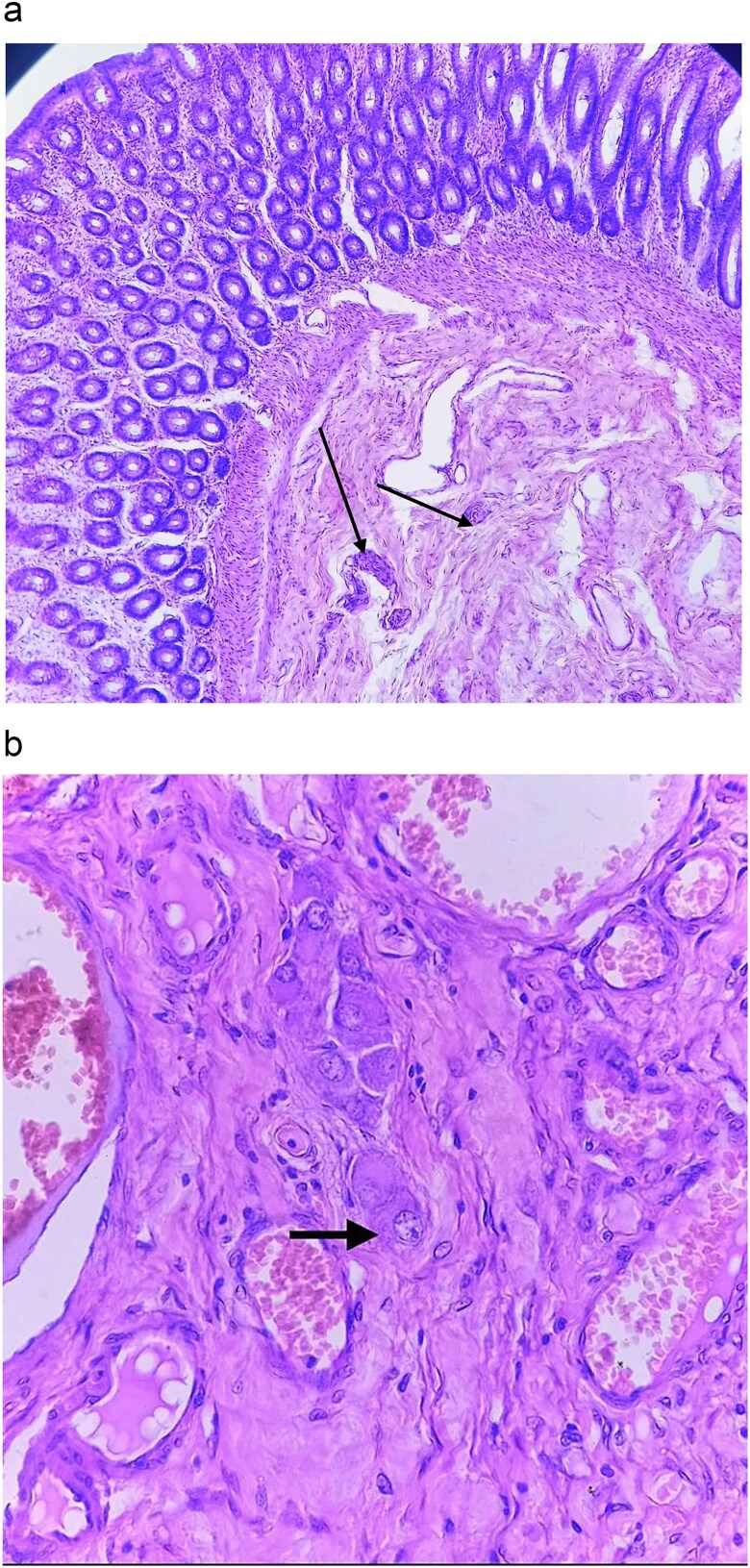
(a) Aganglionic segment with hypertrophied submucosal nerves. (b) Healthy segment at the margin with presence of ganglionic cells.

Histopathology of the distal specimen confirmed the absence of ganglion cells, hypertrophic submucosal nerve trunks, and chronic inflammation (Fig. 3a). The healthy intestinal margin contained ganglionic cells (Fig. 3b). The findings were consistent with HD.

Postoperatively, he required multiple blood transfusions and a single day of intensive care unit (ICU) care. He recovered well and was discharged on the 4th postoperative day with a functioning colostomy.

One month later, the patient re-presented with 2 days of severe abdominal pain, progressive distension, and vomiting. On admission, he was tachycardic (120 bpm), hypotensive (90/60 mmHg), and febrile. The abdomen was grossly distended, tympanic, and tender, with absent bowel sounds. The previously created stoma was nonfunctional. Laboratory findings included hemoglobin 5.4 g/dl, WBC 2500/cu mm (70% neutrophils), CRP 142 mg/L, lactate 2.3 mmol/L, amylase 116 U/L, and ascitic fluid with 3528 cells/cu mm (predominantly neutrophils). Ascitic fluid culture later grew *Escherichia coli*.

**Figure 4:**
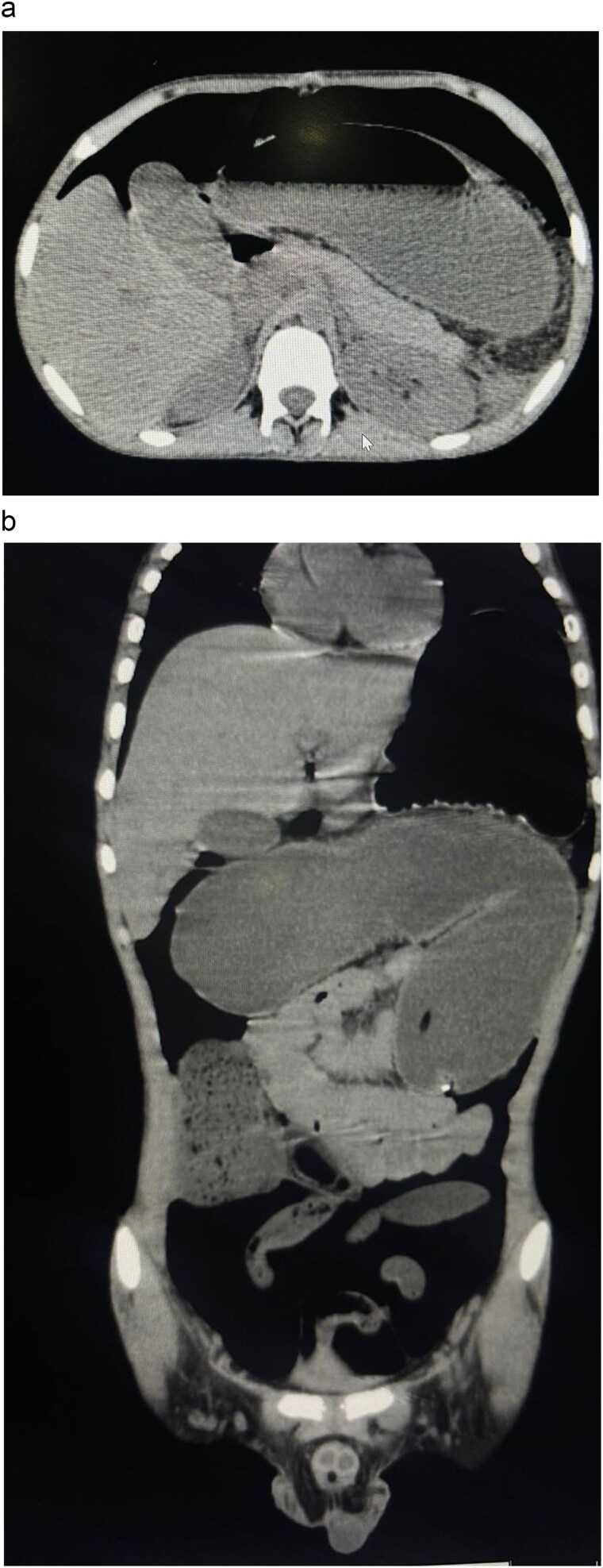
(a) Presence of air-fluid level indicating obstruction. (b) Bowel obstruction with transition point at the stoma.

Axial CT abdomen (Fig. 4a) showed dilated bowel with air-fluid levels suggestive of obstruction/ileus. Contrast-enhanced CT (Fig. 4b) confirmed large bowel obstruction with a transition point at the stoma, gross pneumoperitoneum, and proximal dilatation.

**Figure 5:**
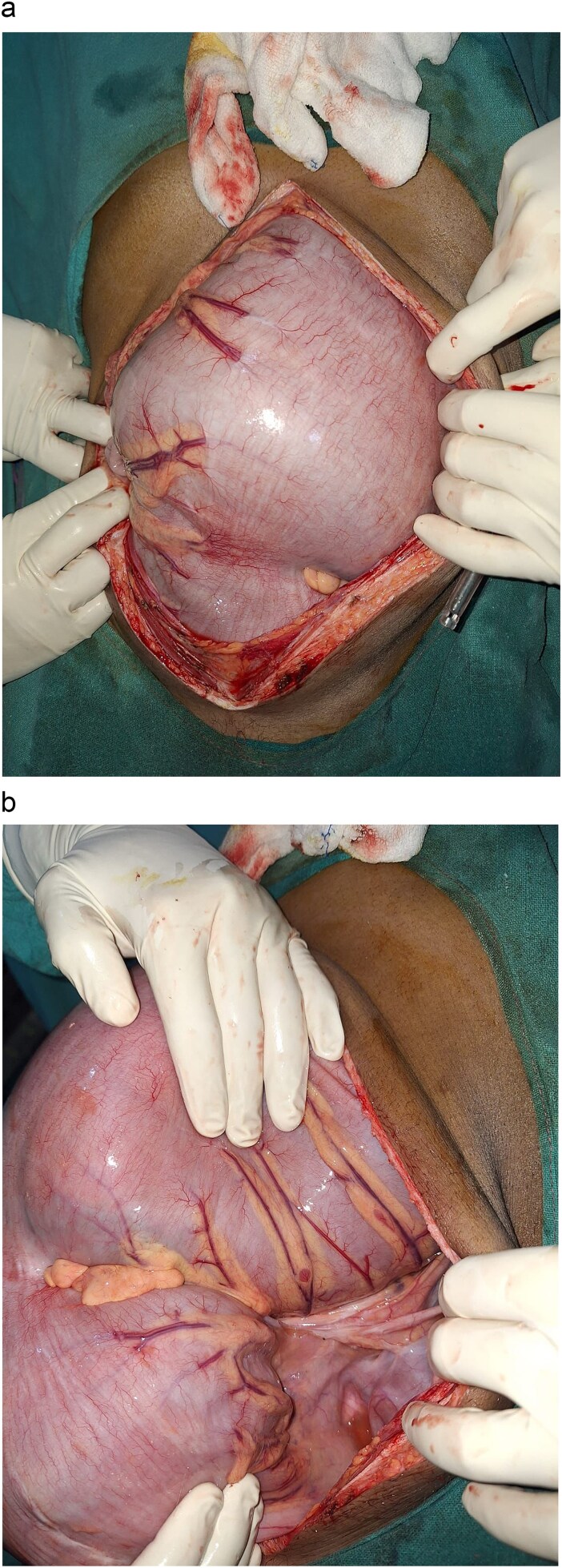
(a and b) Distended colon without volvulus or adhesions.

Urgent exploratory laparotomy revealed a massively dilated colon with serosal tears without volvulus or adhesions (Fig. 5a and b). The distention extended from the stoma to the terminal ileum. The gall bladder was also distended. The patient underwent total colectomy with end ileostomy.

Postoperatively, he required 48 hours of mechanical ventilation, multiple blood transfusions, and multiple days of ICU stay until recovery. After surgery, the sepsis resolved, and the ileostomy output was normalized. He was discharged on Day 10 with a functioning ileostomy. The patient was counseled about possible future ileostomy complications and the need for further surgery. On the 15^th^ day follow-up, the ileostomy was functioning well.

## Discussion

Adult HD is rare and often mistaken for simple chronic constipation. HD is diagnosed and treated before 5 years of age in < 90% of cases [4]. When it presents in adults, it is usually a short-segment or ultra-short-segment type. These small aganglionic parts cause only mild symptoms, so patients manage for many years with laxatives, enemas, and sometimes manual help. The normal colon above compensates for a long time, which delays diagnosis [4, 5].

Our patient had severe constipation since childhood without any major neonatal problems. He controlled it with medications for years. This history matches the typical pattern of adult HD. Adult HD shows male predominance (around 4:1), and patients have hard, infrequent, painful stools [4]. After the first surgery, the remaining colon continued to dilate, the stoma failed, a perforation occurred, and septic shock developed. Complications like bowel perforation, toxic megacolon, sepsis, and even death are well described in delayed or incompletely treated adult cases, often requiring emergency reoperation [6–8].

In young adults with abdominal distension and constipation, doctors usually first suspect colorectal cancer, diverticulitis, or sigmoid volvulus. In Nepal and many parts of South Asia, sigmoid volvulus is one of the most common causes of large bowel obstruction in young males, often linked to chronic constipation or high-fiber diets, so it is a frequent first suspicion before considering HD [5].

The basic problem in HD is missing ganglion cells in the distal bowel, so that segment cannot relax and causes functional obstruction. In mild cases, the patient survives long with medical treatment, but later massive dilatation, obstruction, or perforation can occur [4]. One similar case was reported from Indonesia, where an 18-year-old woman who passed stool only once or twice a month due to ultra-short segment disease, but she did not require surgery [4]. Our patient needed emergency reoperation because of severe complications.

Management of adult HD is difficult due to late presentation and the uncertain length of the affected segment. The first operation was done to preserve remaining colonic function, but progressive dilatation led to perforation and sepsis. We then performed total colectomy with end ileostomy as emergency salvage surgery in response to life-threatening complications [9].

Some limitations were present in our management of the case. Preoperative rectal biopsy or genetic test to confirm the transition zone was not performed due to the limited availability of facilities and acute presentation. Long-term follow-up about bowel function and stoma care is still needed.

This case teaches us that in young patients with very resistant constipation, we must suspect adult HD. A rectal biopsy should be considered in adolescents and young adults with lifelong constipation unresponsive to medical therapy. Early correct diagnosis, surgery planned according to aganglionic level, and strict follow-up are very important to prevent dangerous complications like perforation and septic shock.
